# Supplementary material for: Stacking Model‐Based Classifiers for Dealing With Multiple Sets of Noisy Labels
Source: Biom J. 2025 Mar 12;67(2):e70042. doi: 10.1002/bimj.70042 (PMC11898607; doi:10.1002/bimj.70042)
Supplement: Supplementary file 1 — Supporting Information [file BIMJ-67-e70042-s001.zip › density-based-ensemble-model-main/competitors_real_data/netlab-master/netlab-master/help/rbfprior.htm]

Netlab Reference Manual rbfprior


# rbfprior

## Purpose

Create Gaussian prior and output layer mask for RBF.

## Synopsis

```
[mask, prior] = rbfprior(rbfunc, nin, nhidden, nout, aw2, ab2)
```

## Description

`[mask, prior] = rbfprior(rbfunc, nin, nhidden, nout, aw2, ab2)`
generates a vector
`mask` that selects only the output
layer weights. This is because most uses of RBF networks in a Bayesian
context have fixed basis functions with the output layer as the only
adjustable parameters. In particular, the Neuroscale output error function
is designed to work only with this mask.

The return value
`prior` is a data structure,
with fields `prior.alpha` and `prior.index`, which
specifies a Gaussian prior distribution for the network weights in an
RBF network. The parameters `aw2` and `ab2` are all
scalars and represent the regularization coefficients for two groups
of parameters in the network corresponding to
second-layer weights, and second-layer biases
respectively. Then `prior.alpha` represents a column vector of
length 2 containing the parameters, and `prior.index` is a matrix
specifying which weights belong in each group. Each column has one
element for each weight in the matrix, using the standard ordering as
defined in `rbfpak`, and each element is 1 or 0 according to
whether the weight is a member of the corresponding group or not.

## See Also

`rbf`, `rbferr`, `rbfgrad`, `evidence`

---

**Pages:**
Index


---

Copyright (c) Ian T Nabney (1996-9)
